# Supplementary material for: A whole-animal phenotypic drug screen identifies suppressors of atherogenic lipoproteins
Source: eLife. 2026 Aug 27;14:RP105314. doi: 10.7554/eLife.105314 (PMC13521566; doi:10.7554/eLife.105314)
Supplement: Supplementary file 2 [file elife-105314-code1.zip › JHDL_primary_result/Fendiline HCl/Fendiline HCl_JHDL_7_034_004_2019_06_14_11_12.pdf]

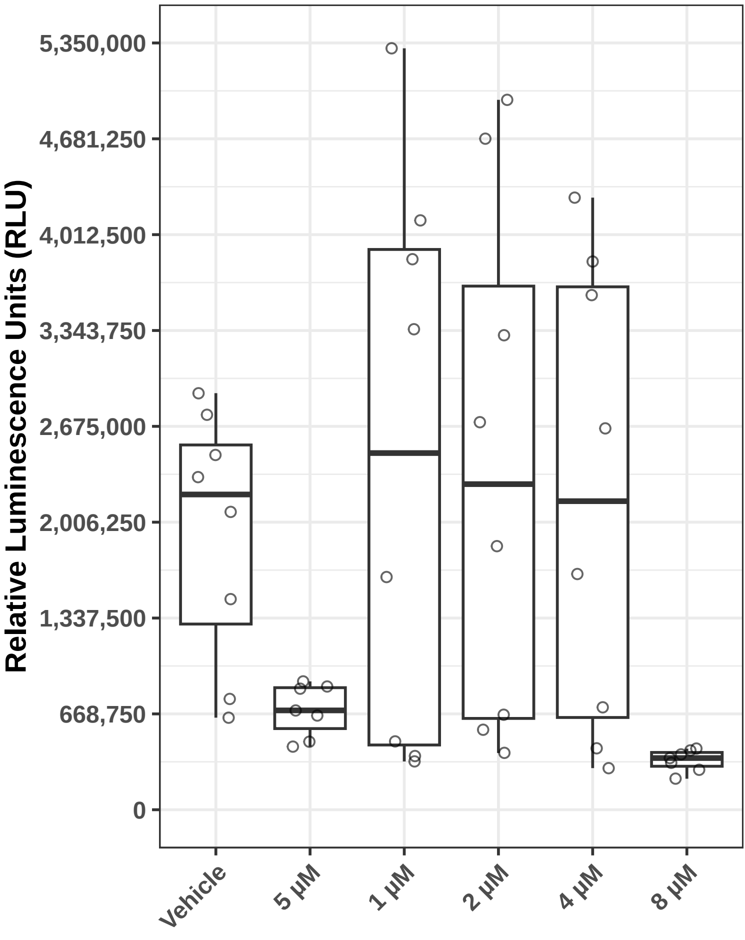

Relative Luminescence Units (RLU)
5,350,000
4,681,250
4,012,500
3,343,750
2,675,000
2,006,250
1,337,500
668,750
0
Vehicle
5 µM
1 µM
2 µM
4 µM
8 µM
